# Cardiac Myocyte *De Novo* DNA Methyltransferases 3a/3b Are Dispensable for Cardiac Function and Remodeling after Chronic Pressure Overload in Mice

**DOI:** 10.1371/journal.pone.0131019

**Published:** 2015-06-22

**Authors:** Thomas G. Nührenberg, Nils Hammann, Tilman Schnick, Sebastian Preißl, Anika Witten, Monika Stoll, Ralf Gilsbach, Franz-Josef Neumann, Lutz Hein

**Affiliations:** 1 Institute of Experimental and Clinical Pharmacology and Toxicology, University of Freiburg, Freiburg, Germany; 2 Universitäts-Herzzentrum Freiburg • Bad Krozingen, Klinik für Kardiologie und Angiologie II, Bad Krozingen, Germany; 3 Universitäts-Herzzentrum Freiburg • Bad Krozingen, Klinik für angeborene Herzfehler und pädiatrische Kardiologie, Hugstetter Straße 55, 79106, Freiburg, Germany; 4 Hermann Staudinger Graduate School, University of Freiburg, Albertstraße 21, 79104, Freiburg, Germany; 5 Core Unit Genomics für Hochdurchsatzgenetik und-genomik an der Medizinischen Fakultät Münster, Westfälische Wilhelms-Universität Münster, Münster, Germany; 6 BIOSS Centre for Biological Signalling Studies, University of Freiburg, Schänzlestr. 18, 79104, Freiburg, Germany; University of Torino, ITALY

## Abstract

**Background:**

Recent studies reported altered DNA methylation in failing human hearts. This may suggest a role for *de novo* DNA methylation in the development of heart failure. Here, we tested whether cardiomyocyte-specific loss of *de novo* DNA methyltransferases *Dnmt3a* and *Dnmt3b* altered cardiac function and remodeling after chronic left ventricular pressure overload.

**Methods:**

Mice with specific ablation of *Dnmt3a* and *Dnmt3b* expression in cardiomyocytes were generated by crossing floxed *Dnmt3a^fl^* and *Dnmt3b^fl^* alleles with mice expressing Cre recombinase under control of the atrial myosin light chain gene promoter. The efficacy of combined *Dnmt3a/3b* ablation (DKO) was characterized on cardiomyocyte-specific genomic DNA and mRNA levels. Cardiac phenotyping was carried out without (sham) or with left ventricular pressure overload induced by transverse aortic constriction (TAC). Under similar conditions, cardiac genome-wide transcriptional profiling was performed and DNA methylation levels of promoters of differentially regulated genes were assessed by pyrosequencing.

**Results:**

DKO cardiomyocytes showed virtual absence of targeted *Dnmt3a* and *Dnmt3b* mRNA transcripts. Cardiac phenotyping revealed no significant differences between DKO and control mice under sham and TAC conditions. Transcriptome analyses identified upregulation of 44 and downregulation of 9 genes in DKO as compared with control sham mice. TAC mice showed similar changes with substantial overlap of regulated genes compared to sham. Promoters of upregulated genes were largely unmethylated in DKO compared to control mice.

**Conclusion:**

The absence of cardiac pathology in the presence of the predicted molecular phenotype suggests that *de novo* DNA methylation in cardiomyocytes is dispensable for adaptive mechanisms after chronic cardiac pressure overload.

## Introduction

Epigenetic mechanisms have evolved as important regulators of development and disease [1,2]. For long time, DNA methylation was mostly considered to be a stable repressive epigenetic mark in somatic cells. However, now DNA methylation in the CpG context is increasingly being recognized as a highly dynamic process [3]. During development, nearly complete DNA demethylation takes place in the pre-implantation embryo which is followed by progressive remethylation starting at the blastocyst stage [4]. High levels of DNA methylation are characteristic for the establishment of somatic cell types with CpG methylation patterns that are highly specific for each cell type [5]. Inactive genomic regions such as heterochromatin tend to be heavily methylated. With regard to promoter regions, it is well established that promoter CpG methylation leads to potent repression of transcription [6]. In gene bodies, loss of CpG methylation has recently been described to correlate strongly with gene transcription in cardiomyocytes [7]. Compared to the established views, postnatal DNA methylation has increasingly emerged to be a dynamic epigenetic feature. This holds true for the CpG context as well as for non-CpG methylation in highly specialized somatic cell types such as neurons and cardiomyocytes [7,8]. In cardiomyocytes, dynamic CpG methylation was predominantly confined to postnatal development but also occurred in experimental heart failure. In failing cardiomyocytes DNA methylation returned to a fetal methylation pattern [7]. In line with these findings, altered CpG methylation was also observed in human heart failure [9,10]. In hearts from patients with end-stage cardiomyopathy undergoing heart transplantation, intra-genic CpG islands displayed higher methylation levels compared to control. In the *DUX4* gene, which shows increased expression in fascioscapulohumeral muscular dystrophy, a hypermethylated gene body went along with reduced mRNA levels in end-stage failing hearts [10]. Yet, it is currently unclear whether the observed changes in DNA methylation are causally linked to mechanisms involved in the development of heart failure.

5’ cytosine methylation in a CpG context can be established by three different DNA methyltransferases. Among them, DNMT1 is considered to be a *maintenance* methyltransferase, thus conserving CpG methylation patterns during DNA replication. Since cardiomyocytes show low rates of DNA synthesis postnatally [11,12], DNMT1 does not emerge as a key player of the observed hypermethylation. Conversely, DNMT3A and DNMT3B are responsible for *de novo* CpG methylation in the absence of DNA replication. Thus, *de novo* CpG methylation in cardiomyocytes is likely caused by DNMT3A and/or DNMT3B. Yet, a general knock-out of *Dnmt3b* is embryonically lethal and *Dnmt3a* homozygous knockout mice die runted at 3 weeks of age [13]. Therefore, we generated mice with cardiomyocyte-specific deletion of *Dnmt3a* and *Dnmt3b*. To gain insight into the relevance of *de novo* CpG methylation in cardiac hypertrophy and failure, we subjected these mice to left ventricular pressure overload by transverse aortic constriction and analysed cardiac gene expression and DNA methylation.

## Methods

### Animal procedures

All animal procedures were conducted following the Guide for the Care and Use of Laboratory Animals published by the National Academy of Sciences 2011 and were approved by the responsible Committee on the Ethics of Animal Experiments (Regierungspräsidium, Freiburg, Germany, permit number: G12/30). All efforts were made to minimize suffering of the animals. Cardiomyocyte-specific double knockout (DKO) mice lacking the catalytic domains of *Dnmt3a* (exon 18) and *Dnmt3b* (exon 19) were obtained by mating *Dnmt3a* ^*flox*^ and *Dnmt3b* ^*flox*^ mice [14,15] with mice expressing a cre recombinase under control of the cardiac atrial myosin light chain promoter (*Myl7*, MLCCre) [16]. For both floxed alleles, severe DNA hypomethylation upon recombination with TNAP-Cre [14] or adenoviral Cre [15] as well as loss of DNMT3A and DNMT3B protein [15] have been shown. Mice with the genotype *Dnmt3a* ^*flox/flox*^, *Dnmt3b* ^*flox/flox*^ without expressing cre recombinase were used as control mice (CTL). To establish left ventricular pressure overload for 4 weeks, 10–12 week old male mice were anaesthetized with 2% (vol) isoflurane in oxygen and underwent transverse aortic constriction (TAC). After partial thoracotomy limited to the upper 2 ribs, a 7.0 silk suture was pulled tightly around both the aortic arch distal to the brachiocephalic trunk and a 27-G hypodermic needle in order to constrict the transverse aorta in a defined way as described previously [17]. 14–16 week-old mice were weighed, sacrificed and heart and lung tissues were removed and dissected for assessment of ventricular and lung weight, respectively.

### Genotyping

DNA was isolated from tail biopsies [7]. Primers for detection of targeted *Dnmt3a*, *Dnmt3b* alleles and MLCCre by PCR are indicated in S1 Table.

### Echocardiography

To monitor cardiac function, transthoracic echocardiography was performed using a Vivid 7 Dimension imaging system (GE Medical Systems, Munich, Germany) equipped with a 14 MHz scanning head (i13L Probe). During the procedure, mice were anesthetized with 2% (vol) isoflurane in oxygen (0.5 l/min) via a respiratory mask and placed in dorsal position on a 37°C heating plate. Left ventricular wall and chamber dimensions as well as heart rate were measured in the M-Mode. Ejection fraction (EF) was calculated as described previously [18]. In TAC and sham mice, the aortic diameter was assessed before and 2 or 4 weeks after surgery.

### Histology

Dissected heart tissue was fixed in 4% paraformaldehyde in phosphate-buffered saline (Roti-Histofix, Carl Roth, Karlsruhe, Germany) over 24 hours and embedded in paraffine after dehydration. Paraffin blocks were cut into 5 μm slices and stained after rehydration with the Picrosirius-Red method (Sigma-Aldrich, Taufkirchen, Germany) or fluorophore-conjugated wheat germ agglutinin (WGA-Alexa Fluor 488 conjugate, Life Technologies, Karlsruhe, Germany). Picrosirius-red stained ventricular sections served for determination of left ventricular interstitial fibrotic area using AxioVision software (Carl Zeiss, Oberkochen, Germany). WGA-stained sections were used for quantification of cardiomyocyte cross-sectional areas after nuclear counterstaining with DAPI (4’,6 diamidino-2-phenylindole, Life Technologies, Karlsruhe, Germany). In each ventricle, ≥ 50 cardiomyocytes were measured using the AxioVision software. Measurements were limited to cardiomyocytes that were cut perpendicular to their long axis at the level of a centered, round cardiomyocyte nucleus.

### Quantitative real-time PCR (qPCR) and microarray analysis

Total RNA was extracted from snap-frozen left ventricular tissue or from cardiomyocytes directly sorted into RLT buffer (Qiagen, Hilden, Germany) with a column-based RNA isolation kit (RNeasy Mini Kit, Qiagen, Hilden, Germany) following the manufacturer’s protocol for fibrous tissue. The RNA amount was measured with a NanoDrop spectrophotometer (Thermo Scientific, Waltham, United States). 200 ng total RNA was reverse transcribed using the QuantiTect Reverse Transcription Kit (Qiagen, Hilden, Germany), followed by SYBR-based quantitative real-time PCR. All mRNA expression levels were normalized to ribosomal protein S29 (*Rps29*) copies according to the ∆Ct method [19]. Sequences of gene-specific primer pairs for *Nppa* and *Myh7* are listed in S1 Table. For microarray analyses with Illumina BeadArrays, total RNA was extracted as mentioned above from left ventricular tissue of sham CTL (n = 4) and DKO mice (n = 4) as well as TAC-operated CTL (n = 6) and DKO mice (n = 6). mRNA isolation and preparation of cRNA were conducted as indicated by the manufacturer (Epicentre TargetAmp-Nano Labeling Kit, Biozym Scientific, Hessisch Oldendorf, Germany). After preparation of cRNA, array analyses were carried out at the Core Unit Genomics für Hochdurchsatzgenetik und-genomik of the Medical School Münster (Münster, Germany). For data analysis, Illumina GenomeStudio V2010.3 was used (pFDR < 0.05, fold change > 1.5). All microarray data were deposited at the NCBI database (GEO accession number GSE68518).

### Cell sorting of cardiomyocytes and cardiomyocyte nuclei

Hearts of sacrificed adult male mice were immediately removed *in toto* and digested by retrograde perfusion with 0.8 mg/ml collagenase B (Roche, Mannheim, Germany) and 3 μg/ml trypsin (Sigma-Aldrich, Taufkirchen, Germany) solved in Tyrode’s buffer containing 25 mM butanedione monoxime and 2 mM CaCl_2_. After 12 minutes, the enzymatic digestion was stopped by adding Tyrode’s buffer supplemented with 5% fetal calf serum, 25 mM butanedione monoxime and 2 mM CaCl_2_. During the whole procedure, all solutions and the cell suspension were kept at 37°C. Digested hearts were gently dissected and filtered through a 100 μm cell strainer (BD, Heidelberg, Deutschland). Cells were then washed in phosphate-buffered saline containing 1 mM EDTA and incubated with 7-AAD (Life Technologies, Karlsruhe, Germany). Viable cardiomyocytes, representing the largest cell fraction with simultaneous absence of 7-AAD staining, were sorted with a S3 cell sorter (BioRad, Munich, Germany). Enrichment of cardiac myocytes was confirmed by qPCR for cell type-specific expression marker genes of cardiomyocytes, endothelial cells and fibroblasts. Cardiomyocyte nuclei were isolated from male hearts as described previously [7,20]. Cardiomyocyte nuclei were stained by an antibody directed against PCM-1 (HPA023370, Sigma-Aldrich, Taufkirchen, Germany), followed by an Alexa488-conjugated secondary anti-rabbit antibody (Life Technologies, Karlsruhe, Germany) for 30 min and 7-AAD (1:500, Invitrogen, Karlsruhe, Germany). 7-AAD^+^, Alexa-488^+^ cardiomyocyte nuclei were directly sorted into RLTplus buffer (Qiagen, Hilden, Germany) using the S3 cell sorter.

### Assessment of recombination in cardiomyocyte nuclei

From sorted nuclei, genomic DNA was extracted using the AllPrep DNA/RNA Mini Kit (Qiagen, Hilden, Germany). To evaluate recombination efficiency, qPCR was performed with 2.5 ng of genomic DNA and primers for amplicons spanning exon-intron borders in *Dnmt3a* and *Dnmt3b* genes. *Sry* served as reference gene. The percentage of recombination was calculated using the ∆Ct method [19].

### Pyrosequencing

Pyrosequencing assays were designed with the PyroMark assay design software 2.0 for genes that were 1) upregulated in the array analysis between CTL and DKO mice both under sham and TAC conditions and 2) had highest expression after TAC. The designed assays contained 3–4 single CpGs that are located in the respective promoter regions (TSS ± 500 bp). 100 ng genomic DNA from FACS-sorted cardiomyocyte nuclei was converted by bisulfite using the EZ DNA Methylation Kit (D5001, Zymo Research, Freiburg, Germany). 5–10 ng of bisulfite-converted DNA was then amplified with the PyroMark PCR Kit (Qiagen, Hilden, Germany) in an approach using three primers including a biotinylated universal primer for subsequent streptavidin-based DNA capture [21]. After validation of PCR products by gel electrophoresis, amplicons were pyrosequenced using PyroMark Gold Q24 Reagents (Qiagen, Hilden, Germany) on a PyroMark Q24 instrument [22] and quantified with the PyroMark Q24 software (Qiagen, Hilden, Germany). Primer sequences are listed in S1 Table.

### Statistical Analysis

If not stated otherwise, data are expressed as mean ± standard error of the mean (SEM). For comparison between two groups, Mann-Whitney-U-test was used. For multiple comparisons, Kruskal-Wallis-test with Dunn’s multiple comparison post-hoc test was applied. P values < 0.05 were considered as significant. All statistical analyses except for the Illumina BeadArray analysis were performed with GraphPad Prism version 4.01 (GraphPad Software, San Diego, United States).

## Results

### Cardiomyocyte-specific deletion of *Dnmt3a* and *Dnmt3b*

**Fig 1:**
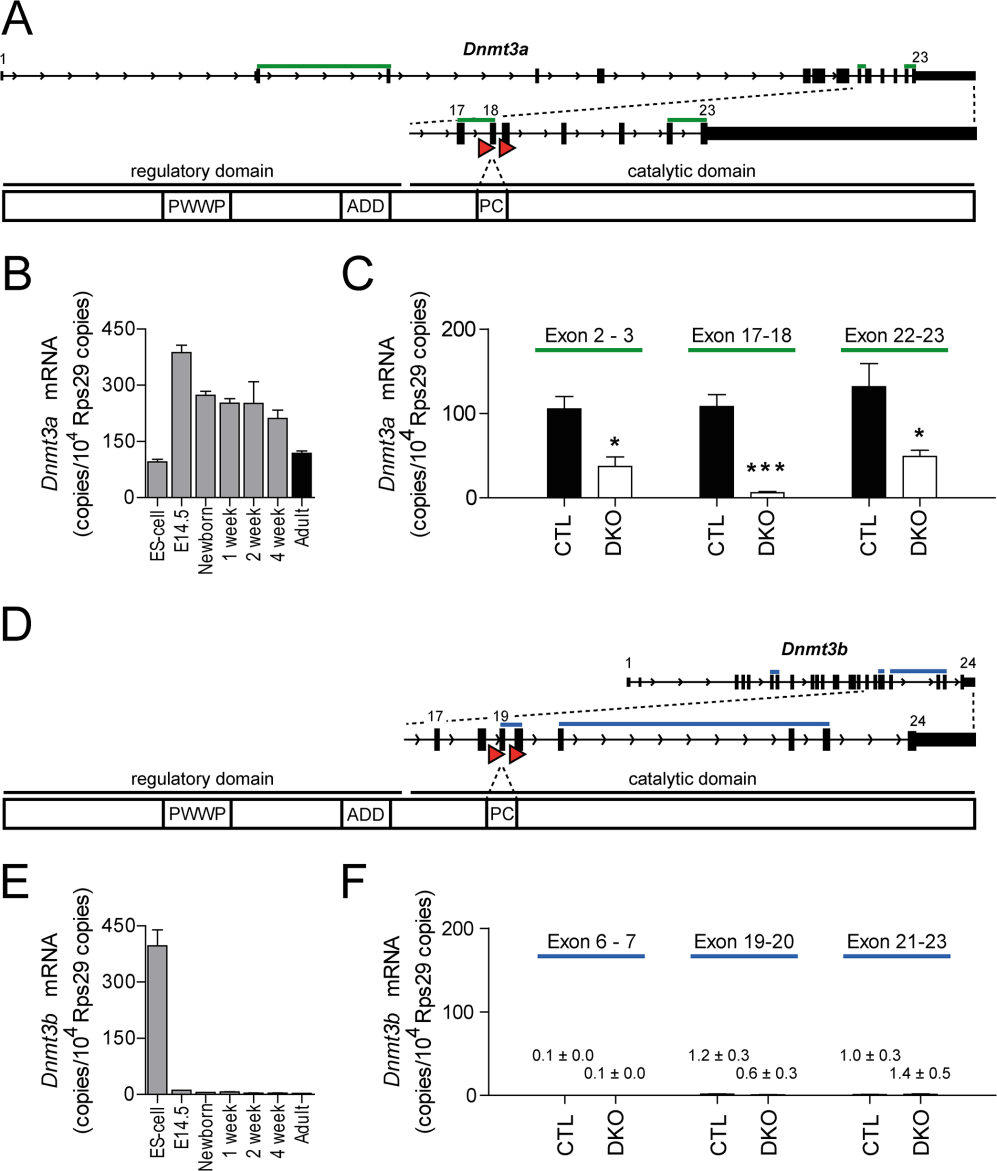
Cardiomyocyte-specific deletion of *Dnmt3a* and *Dnmt3b*. **(A, D)** Visualization of the *Dnmt3a* (A) and *Dnmt3b* (D) gene loci and protein domain structure. The prolylcysteinyl (PC) dipeptide providing thiolate at the active catalytic site is encoded in exon 18 of *Dnmt3a* (A) and in exon 19 of *Dnmt3b* (D). LoxP sites are represented by red triangles. Green (A) and blue (D) bars indicate intron-spanning primers for three different amplicons for determination of *Dnmt3a* (A) and *Dnmt3b* (D) transcripts. **(B, E)** *Dnmt3a* (B) and *Dnmt3b* (E) mRNA levels in embryonic stem (ES) cells (n = 3) and in cardiac tissue at the indicated time points (n = 4–5). **(C, F)** *Dnmt3a* (C) and *Dnmt3b* (F) mRNA levels in sorted cardiomyocytes. *Dnmt3* copies/10^4^ *Rps29* copies are visualized for the intron-spanning amplicons as indicated. n = 5 for control (CTL, filled bars), n = 4 for DKO (open bars) cardiomyocytes, *p < 0.05, ***p < 0.001.

**Fig 2:**
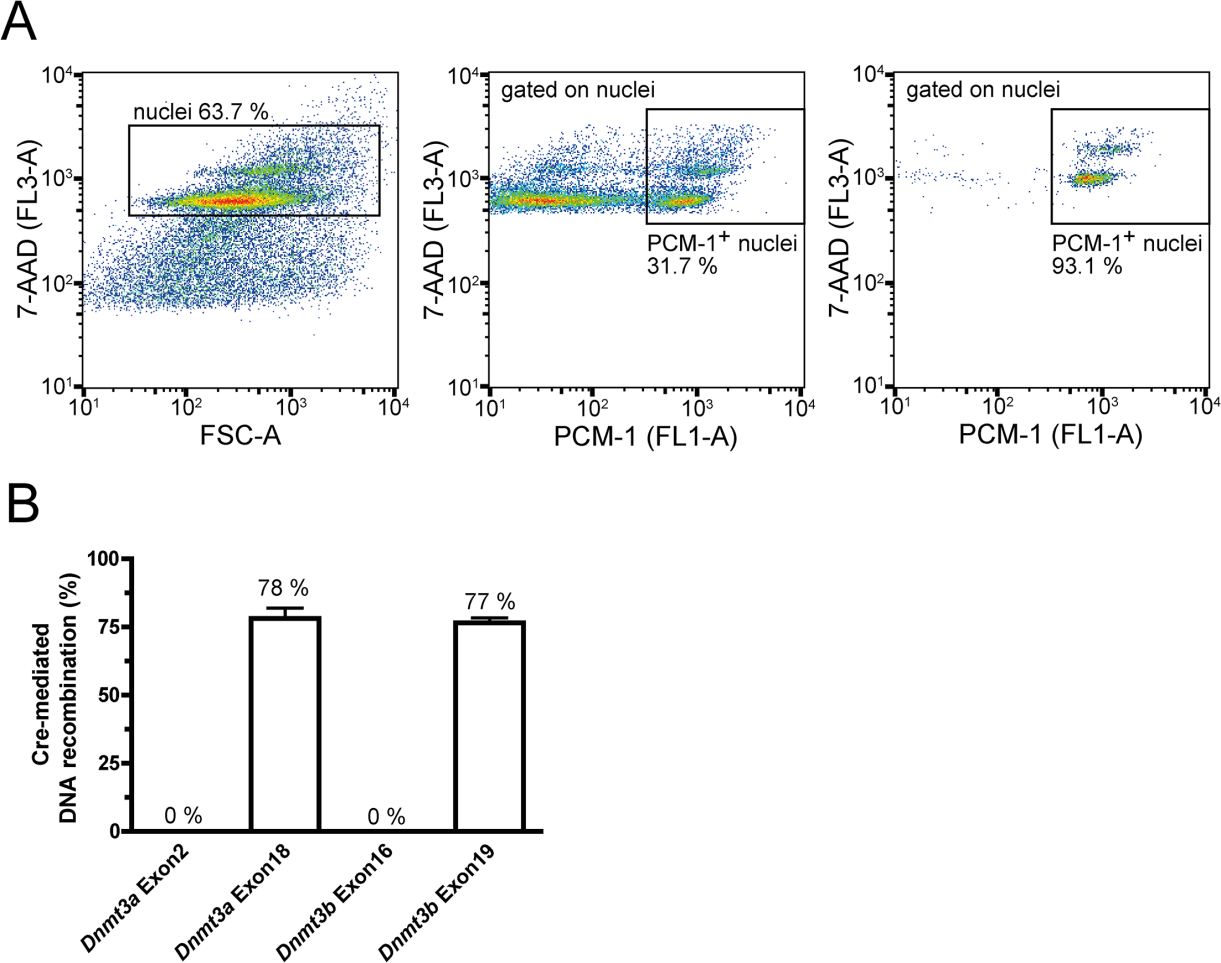
Cardiomyocyte-specific Cre-mediated recombination. **(A)** Representative flow cytometry plots depicting the gating strategy for sorting of cardiomyocyte nuclei. Positive 7-AAD (left panel) and PCM-1 (middle panel) staining identified cardiomyocyte nuclei. Purity of sorted cardiomyocyte nuclei is demonstrated in the right panel. FSC represents forward scatter. **(B)** Recombination rates in sorted male cardiomyocyte nuclei. Recombination rates were calculated from relative expression of respective *Dnmt3* exons compared to *Sry* levels (n = 6).

In a breeding of homozygous floxed *Dnmt3a* ^*fl*^ and *Dnmt3b* ^*fl*^ mice with one paternal MLC2aCre allele, litters contained DKO mice that developed normally and were fertile. The loxP sites in the *Dnmt3a* and *Dnmt3b* genes were located up- and downstream of exon 18 or exon 19, respectively (Fig 1A and 1D). Expression of *Dnmt3a* and *Dnmt3b* showed distinct time courses in embryonic stem cells and in the developing heart (Fig 1B and 1E). Cardiac *Dnmt3a* expression peaked during embryonic development but remained strongly expressed in adult mouse hearts (Fig 1B), whereas *Dnmt3b* was almost exclusively expressed in embryonic stem cells (Fig 1E). The effectiveness of cardiomyocyte-specific ablation of *Dnmt3a* and *Dnmt3b* was determined at multiple levels. In order to validate the deletion of *Dnmt3a* and *Dnmt3b* on the transcriptional level, RNA was extracted from FACS-sorted cardiomyocytes of DKO and control mice. In cardiomyocytes, *Dnmt3a* mRNA levels at the deleted exon 18 were almost completely absent (-94%, p = 0.0004, Fig 1C). In addition, mRNA levels at exons upstream or downstream of the floxed exon 18 were significantly lower as well (Fig 1C). *Dnmt3b* expression in cardiomyocytes was at the lower limit of detection and thus further reduction by cardiomyocyte-specific knockout could not be detected (Fig 1F). In order to assess the frequency of recombination, cardiomyocyte nuclei were isolated and purified by FACS sorting (Fig 2A). After identification by 7-AAD staining (left panel), nuclei were classified into PCM-1^-^ non-myocyte and PCM-1^+^ cardiomyocyte nuclei (middle panel). Purity of sorted cardiomyocyte nuclei was 94.0% ± 0.7%. The recombination frequency in sorted cardiomyocyte nuclei was 78.1% ± 3.9% at exon 18 of *Dnmt3a* and 76.5% ± 1.9% at exon 19 of *Dnmt3b* (Fig 2B).

### Cardiac phenotyping of sham and TAC mice

**Fig 3:**
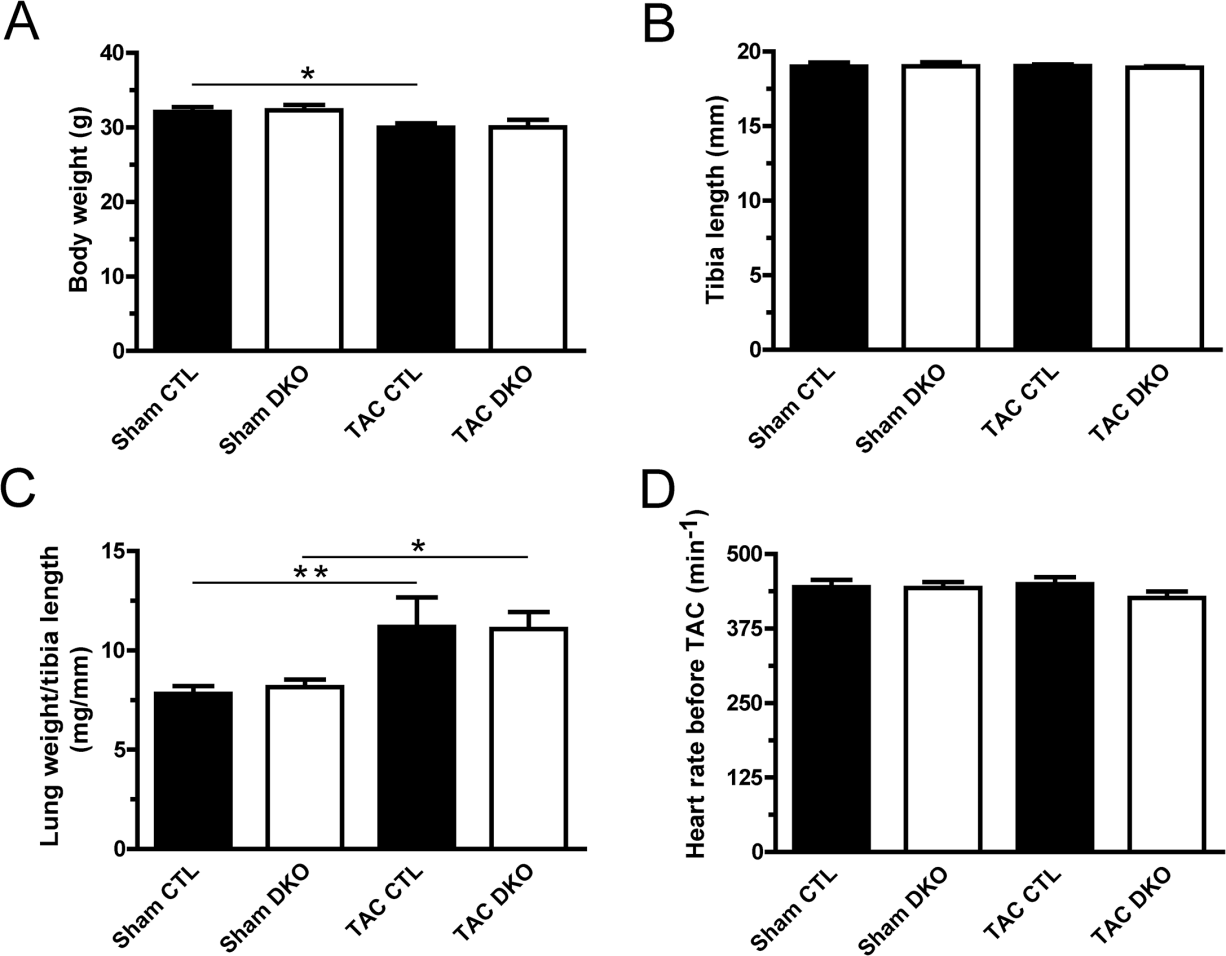
General characteristics of sham and TAC-operated mice. Mice were sacrificed and **(A)** body weight and **(B)** tibia length were measured. **(C)** Lung weight to tibia length ratio was calculated to assess possible pulmonary congestion. **(D)** Heart rate was determined in short axis M-mode measurements. Sham CTL (n ≥ 9), sham DKO (n ≥ 5), TAC CTL (n ≥ 9), TAC DKO (n ≥ 9). * p < 0.05, ** p < 0.01.

**Fig 4:**
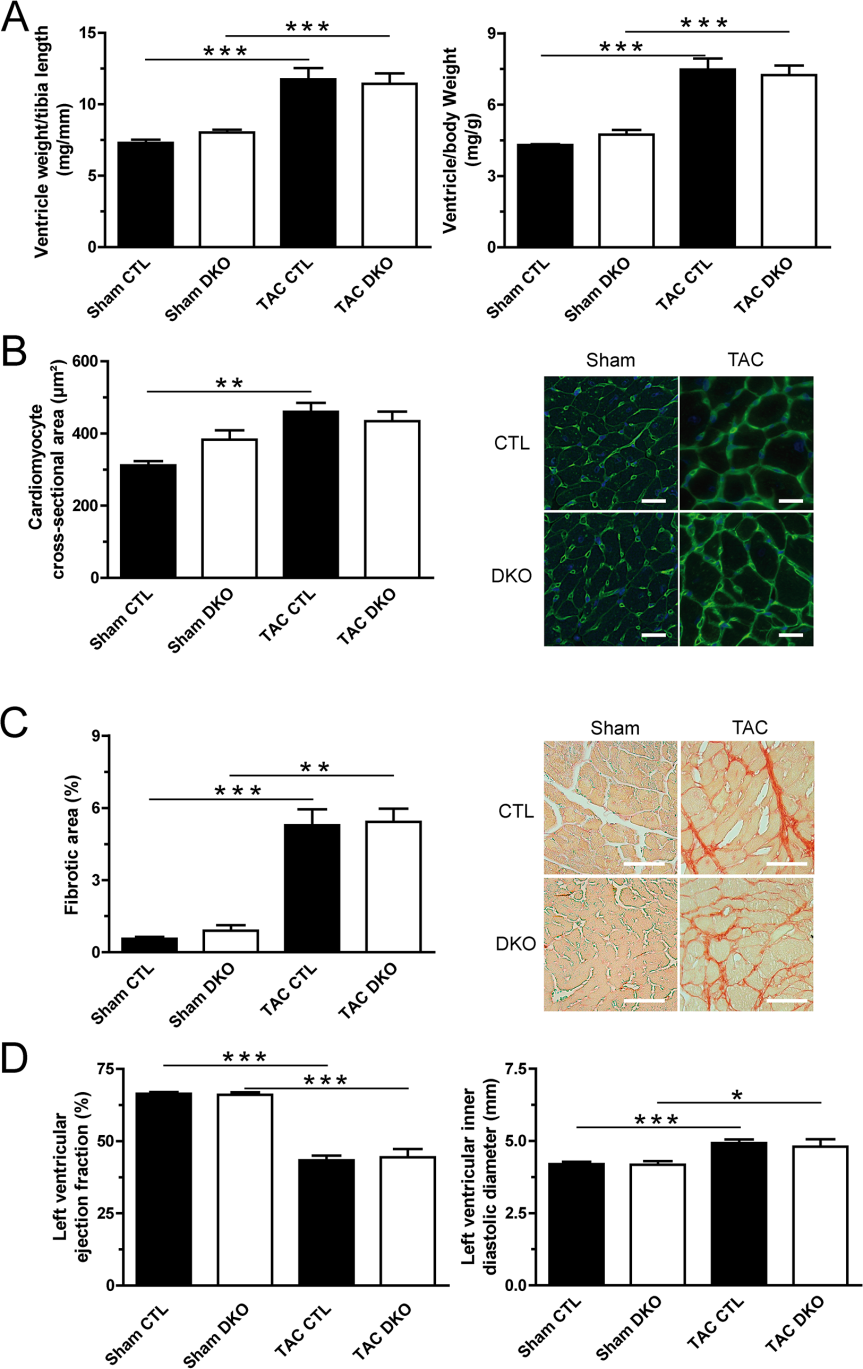
Cardiac phenotyping in sham- and TAC-operated mice. **(A)** Ratios of ventricle weight to tibia length (left panel) and ventricle weight to body weight (right panel) are depicted. n ≥ 6 for control (CTL, filled bars), n ≥ 6 for DKO (open bars) mice, ***p < 0.001. **(B)** Cardiomyocyte cross-sectional area (left panel) assessed after wheat-germ agglutinin staining (right panel, representative sections, scale bar indicates 20 μm), **p < 0.01. **(C)** Cardiac fibrosis (left panel) assessed after picro-sirius red staining (right panel, representative sections, scale bar indicates 50 μm). **(D)** Cardiac function assessed by echocardiography. Left ventricular ejection fraction (left panel) was calculated from short axis M-mode measurements. Left ventricular dimensions are represented by left ventricular inner diastolic diameter (right panel). *p < 0.05, ***p < 0.001.

**Fig 5:**
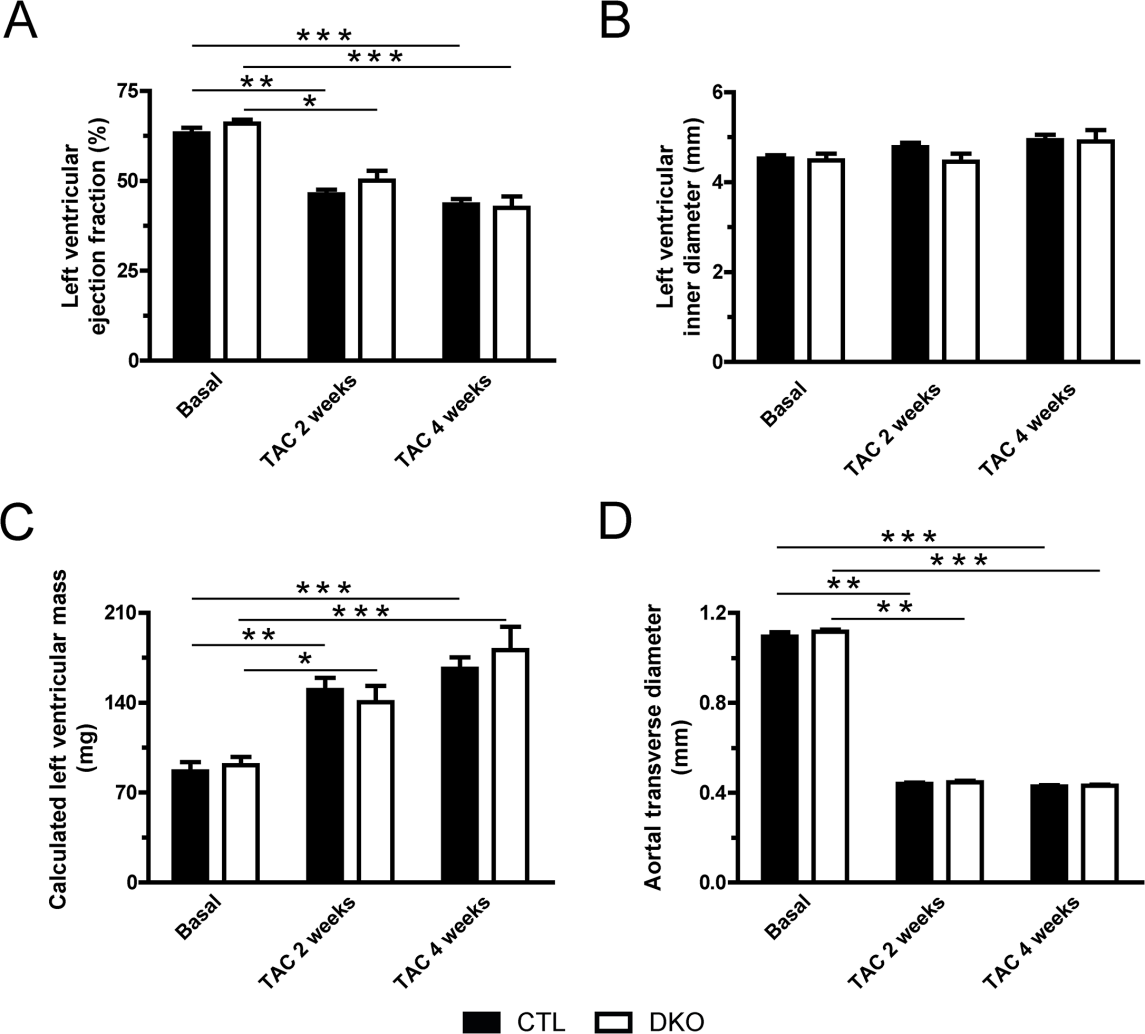
Echocardiography of TAC-operated mice. Echocardiography was performed to assess cardiac parameters of n ≥ 10 control (CTL, filled bars) and n ≥ 9 DKO (open bars) mice before, 2 weeks after, and 4 weeks after TAC. **(A)** Left ventricular ejection fraction was assessed from short axis M-mode measurements. **(B)** Left ventricular dimensions are represented by left ventricular inner diastolic diameter. **(C)** Troy formula was used to calculate left ventricular mass. **(D)** Aortic transverse diameter was measured in the short axis B-mode. * p < 0.05, ** p < 0.01, *** p < 0.001.

The cardiac phenotype was assessed by several means in sham- and TAC-operated mice 4 weeks after the procedure. Ventricular weight to body weight (VW/BW) and ventricular weight to tibia length (VW/TL) ratios were determined for all animals (Figs 3 and 4). Pressure overload showed induction of cardiac hypertrophy by increased VW/BW- and VW/TL-ratios as well as increased cardiac myocyte cross-sectional areas (Fig 4A and 4B). This was accompanied by increased cardiac fibrosis (Fig 4C) and by features of heart failure such as impaired systolic left ventricular ejection fraction and ventricular dilatation (Fig 4D). However, ablation of *Dnmt3a* and *Dnmt3b* expression in cardiomyocytes did not lead to significant differences in any echocardiographic parameter neither in sham nor in TAC animals (Figs 4D and 5).

### Gene expression analyses

**Fig 6:**
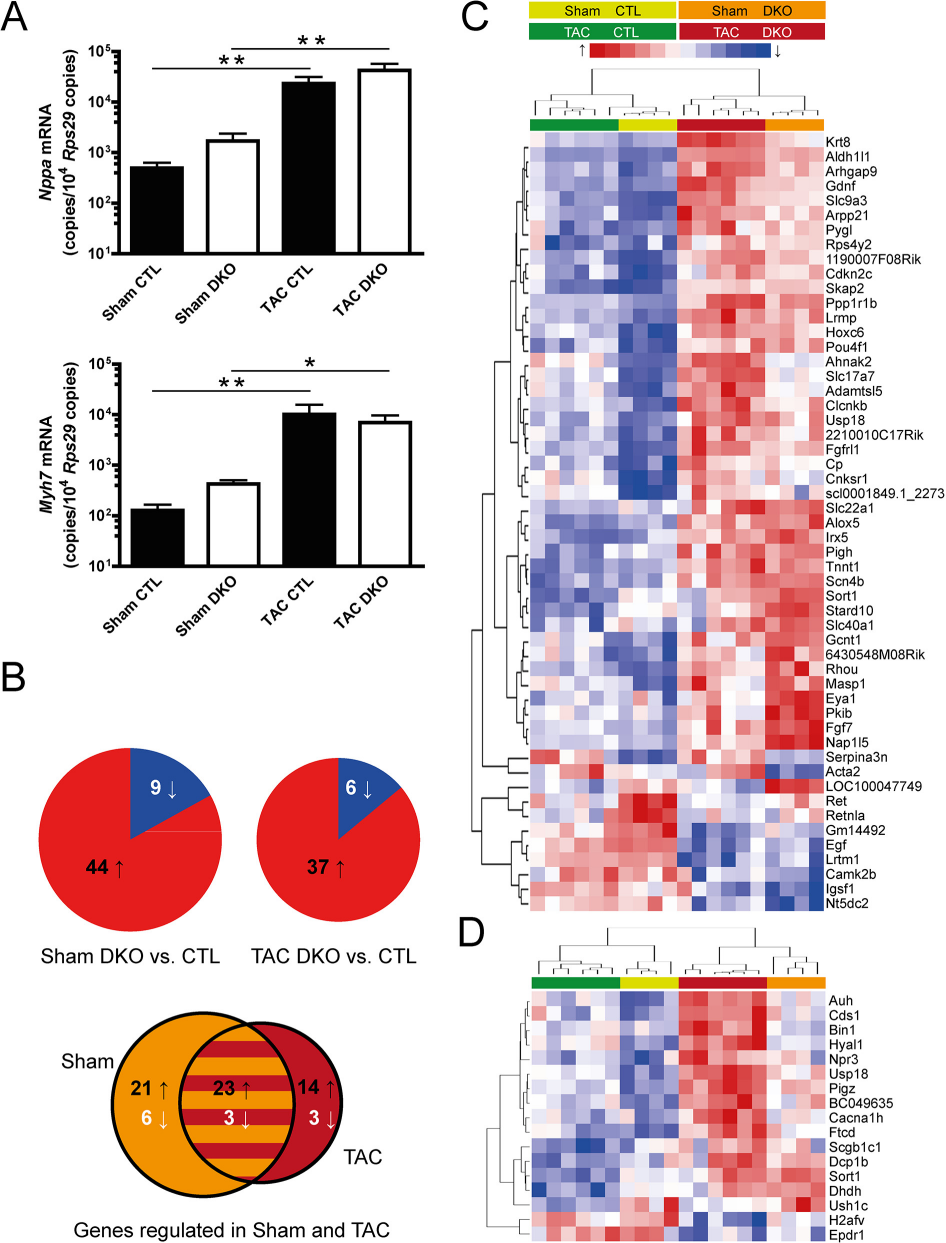
Gene expression analysis. **(A)** Assessment of ventricular mRNA expression by quantitative real-time PCR of atrial natriuretic peptide (*Nppa*, upper panel) and β-myosin heavy chain (*Myh7*, lower panel). n ≥ 6 for control (CTL, filled bars), n ≥ 6 for DKO (open bars) mice, **p < 0.01, *p < 0.05. **(B, C)** Genome-wide assessment of ventricular mRNA expression by Illumina bead chip array analysis in sham- and TAC-operated CTL and DKO mice. n = 4 for each genotype under sham conditions, n = 6 for each genotype under TAC conditions. **(B)** Pie charts indicate the number of up- and downregulated genes between genotypes under sham conditions (left pie chart) and under TAC conditions (right pie chart). The Venn diagram displays the number of genes regulated both under sham- and TAC-conditions and the overlapping proportion. **(C, D)** Heatmaps of the differentially regulated genes. Individual expression values are displayed after unsupervised complete linkage clustering. **(C)** Genes differentially regulated between CTL and DKO under sham conditions. **(D)** Genes with differential regulation between CTL and DKO genotypes only under TAC conditions.

Expression of genes with known induction in heart hypertrophy and failure such as *Nppa* and *Myh7* was assessed by qPCR (Fig 6A). These genes were analyzed as general markers of heart failure. Especially the *Myh7* locus is completely demethylated in adult cardiomyocytes [7]. Thus, we did not expect differences in expression between genotypes. Both genes were strongly upregulated after pressure overload, but again no significant differences were noted between the genotypes. To gain a more detailed insight into genome-wide transcriptional changes, we performed mRNA array analysis for DKO and control mice, both for sham and TAC conditions. In the sham group, 44 genes were upregulated in DKO mice while 9 genes were downregulated compared to control mice (Fig 6B, left pie chart). Results after TAC operation were largely similar with 37 genes showing increased and 6 genes showing decreased expression in DKO mice (Fig 6B, right pie chart). Of these 43 genes, 26 (60%) were similarly regulated in both sham and TAC-operated mice (Fig 6B, Venn diagram). Next, genes that were regulated between genotypes under sham conditions were clustered together with respective gene expression values after TAC (Fig 6C). The resulting heatmap demonstrated that the genotype rather than the disease state had a strong influence on clustering. For those 17 genes that exhibited differential expression exclusively after TAC, a separate heatmap was generated (Fig 6C). All genes showed similar direction of regulation in sham mice.

### Reduced DNA methylation and enhanced RNA expression

**Fig 7:**
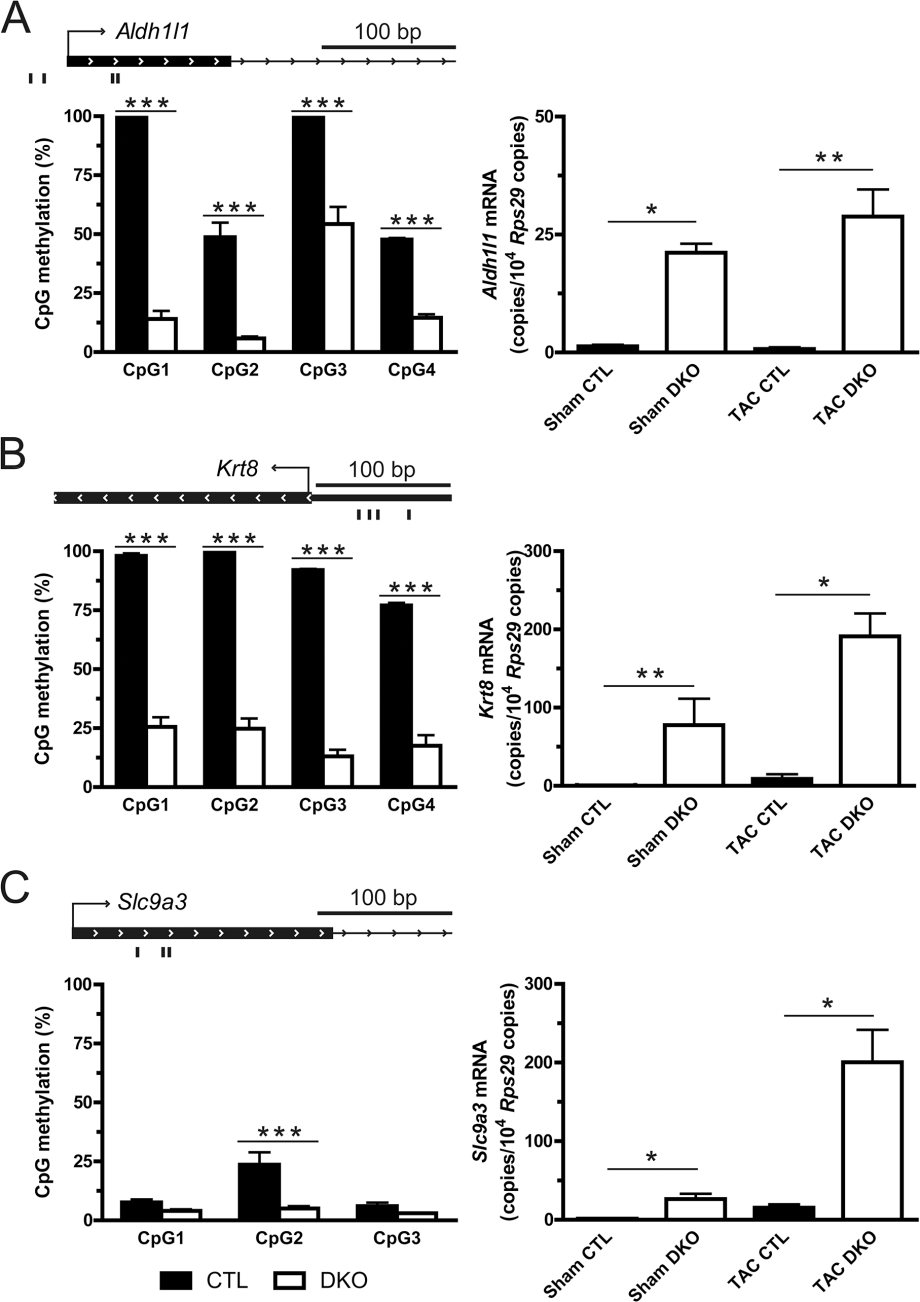
Reduced promoter CpG methylation and enhanced RNA expression. Left panels display the methylation levels of single CpGs in the promoter regions (TSS ± 500 bp) of indicated genes under sham conditions. The black bars below the genomic loci indicate the location of the respective CpGs analyzed by pyrosequencing. Right panels depict mRNA levels for the respective genes under sham and TAC conditions assessed by quantitative real-time PCR. **(A)** Aldehyde dehydrogenase 1 family, member L1 (*Aldh1l1*), **(B)** keratin 8 (*Krt8*) and **(C)** solute carrier family 9, subfamily A (*Slc9a3*). * p < 0.05, ** p < 0.01, *** p < 0.001.

In order to analyze the effect of *Dnmt3a/3b* ablation on DNA methylation, three genes that showed increased expression in DKO mice and had the highest additional increase after TAC, were selected as candidate genes for pyrosequencing. In analyses of sorted cardiomyocyte nuclei from sham-operated hearts, 9 out of 11 single CpG sites were significantly hypomethylated in DKO compared to control mice confirming the suppression of *de novo* DNA methylation (Fig 7, left panels). In line with the array analysis of sham mice, gene expression of *Aldh1l1*, *Slc9a3* and *Krt8* was significantly higher in DKO mice. Under TAC conditions, changes in gene expression were again in line with the array results (Fig 7, right panels).

## Discussion

Heart failure remains a disease with high morbidity and mortality. Currently, pharmacologic approaches are mostly limited to protect against negative sequelae of neurohumoral compensatory mechanisms. While approaches targeting epigenetic features have been proposed for cancer therapies [23–25], many questions need to be answered before such strategies might be used to treat heart failure. Among others, CpG hypermethylation observed in certain cancer entities should be ascertained to play a role in heart failure as well. Concordantly, deletion of hypermethylation capacity might reveal beneficial effects in experimental heart failure. With regard to CpG hypermethylation, proof of such hypermethylation patterns in failing human hearts has been reported in two independent studies [9,10].

In the current study, we therefore focused on enzymes that contribute to increased CpG methylation such as the *de novo* methyltransferases *Dnmt3a* and *Dnmt3b*. For the first time, we examined the effects of combined cardiomyocyte-specific deletion of *Dnmt3a* and *Dnmt3b* in the setting of experimental heart hypertrophy and failure. As the main finding, we did not find significant differences in cardiac function and structure between DKO and control mice neither at baseline nor after pressure overload. Nevertheless, we detected an upregulation of genes that was accompanied by profound reduction of CpG methylation in the promoter regions of these genes. This demonstrates that the applied strategy targeting *Dnmt3a* and *Dnmt3b* indeed manifests in deficient *de novo* CpG methylation. Next to this proof of the molecular phenotype, we made considerable efforts isolating highly purified cardiomyocytes from mouse hearts in order to confirm the effective, cardiomyocyte-specific knockout. In these cardiomyocytes, transcripts of the *Dnmt3a* and *Dnmt3b* catalytic domains were virtually absent. Therefore, we conclude that *de novo* CpG methylation in cardiomyocytes is dispensable for normal heart function and adaptation to disease.

In a recent report, ablation of *Dnmt3b* in adult mice using a tamoxifen-inducible Cre recombinase led to spontaneous cardiomyopathy within two months after tamoxifen induction [26]. However, ablation of *Dnmt3a* and *Dnmt3b* in the present study did not show any signs of spontaneous contractile dysfunction or structural changes. In this model, Cre induction occurred early during cardiac development and may trigger epigenetic mechanisms which compensate for the loss *de novo* DNA methylation during prenatal development. Thus, further studies are required to test, whether loss of Dnmt3a/b differently affects cardiomyocyte function dependent on the timing of Dnmt3a/b ablation.

The hypermethylation of CpG islands which was observed in human heart failure might not be due to catalytic activity of *Dnmt3a* or *Dnmt3b* in cardiomyocytes. Rather, dynamic CpG methylation of non-myocytes may explain the reported differential methylation patterns in the human studies. Recently, our group has demonstrated that alterations in tissue DNA methylation correlate with cardiac tissue composition in pressure overload-induced experimental heart failure [7]. Whole-genome bisulfite sequencing of isolated cardiomyocyte nuclei showed less pronounced changes in CpG methylation after TAC whereas the larger part of altered cardiac DNA methylation was attributed to a decrease of cardiomyocyte content in heart failure [7]. Indeed, this loss of cardiomyocytes and replacement by fibrotic tissue is considered to be a hallmark in the development of heart failure. Therefore, a large part of the differences observed in previous human DNA methylation studies might be due to changes in the tissue composition.

### Limitations

Certainly, this study has several limitations. First, the genetic deletion in our mouse model was confined to the catalytic domains of *Dnmt3a* and *Dnmt3b*. This targeting strategy of *Dnmt3a* and *Dnmt3b* genes has been shown to ablate expression of DNMT3A and DNMT3B protein in mouse embryonic feeder cells [14,15]. It remains possible that truncated DNMT3 isoforms without catalytic function retain other regulatory functions in cardiomyocytes. Yet, the genetic deletion strategy in our study intentionally inhibited specifically *de novo* CpG methylation and thus decreased the possibility of undesired, off-target genetic deletions.

In this study, we demonstrated absence of *de novo* methylation only for a limited number of CpGs located in promoter regions. Concerning other genomic locations, our group has recently shown absence of postnatal gene body remethylation using the same conditional knockout model [7]. Further studies in this model assessing global methylation patterns will be needed to better determine the genome-wide profile of *de novo* CpG methylation mediated by DNMT3A and DNMT3B. Second, our model with combined embryonic deletion of *Dnmt3a* and *Dnmt3b* precludes testing whether one DNMT3 iso-enzyme might compensate for the loss of the other iso-enzyme.

Third, the disease model used in our study leads to rapid onset of cardiac hypertrophy with progression to cardiac dysfunction within four weeks. In contrast, human heart failure develops in most cases over years. Thus, it is conceivable that observed changes in cardiac CpG methylation might evolve very slowly and are not optimally gauged in the TAC model. Nevertheless, TAC has long been established as a valid model for induction of experimental heart hypertrophy and failure [27]. Despite its experimental nature, samples from failing hearts after TAC also bear advantages compared to samples obtained from patients. The clinical samples are usually from patients taking heart failure medications which is absent in TAC. Also, variability of many other patient characteristics such as comorbidities is inherently higher in patients than in inbred mice.

### Conclusions

For the first time, *de novo* CpG methylation capacity was deleted in murine cardiomyocytes. The absence of a cardiac pathology in the presence of the predicted molecular phenotype suggests that *de novo* CpG methylation is dispensable for adaptive mechanisms in heart failure. In light of our data, further studies that examine cardiomyocyte-specific CpG methylation in human heart failure are urgently needed.

## Supporting Information

S1 Table Primer sequences. The listed primers were used for RNA quantification by qPCR, for genotyping, for assessment of DNA recombination in purified cardiomyocyte nuclei and pyrosequencing of bisulfite-converted DNA.
(XLSX)
